# CoSafe: A Cooperative V2V Perception Framework with LLM Reasoning for Hazard Detection on Real Dashcam Data

**DOI:** 10.3390/s26185797

**Published:** 2026-09-13

**Authors:** Iosif-Alin Beti, Paul-Corneliu Herghelegiu, Constantin-Florin Caruntu

**Affiliations:** 1 Department of Automatic Control and Applied Informatics, Gheorghe Asachi Technical University of Iasi, 700050 Iasi, Romania; iosif-alin.beti@student.tuiasi.ro (I.-A.B.); caruntuc@ac.tuiasi.ro (C.-F.C.); 2 Department of Computer Science and Engineering, Gheorghe Asachi Technical University of Iasi, 700050 Iasi, Romania

**Keywords:** cooperative perception, vehicle-to-vehicle communication, spatial–temporal matching, occlusion resolution, fail-safe validation, real-world data, ADAS

## Abstract

Cooperative perception through vehicle-to-vehicle (V2V) communication can resolve occlusions that single-vehicle systems cannot overcome, yet existing frameworks rely on simulated environments and expensive multi-sensor platforms. This paper presents CoSafe, a cooperative perception and reasoning framework built on real-world data acquired from dashboard cameras with integrated GPS. CoSafe extends a previously validated image-based positioning algorithm by adding YOLOv8 object detection, cooperative state fusion, Chain-of-Thought reasoning using a Large Language Model, and a deterministic rule-based safety validation layer. The core contribution is a spatial–temporal vehicle matching framework that associates frames captured from overlapping geographic locations at different timestamps, enabling cooperative hazard reasoning across asynchronous and partially observable vehicle streams. This paper also introduces the Cooperation Gain metric to quantify the proportion of cases in which hazard detection depends on inter-vehicle information sharing. On a real-world occluded-pedestrian scenario, cooperation provides an advance warning of at least 1.73 s before the following vehicle’s own detector registers the pedestrian, with a Cooperation Gain of 0.533 measured against manual human annotation. An ablation across single-vehicle, rule-only, and LLM-only configurations isolates the contribution of each component, and a negative-scenario test yields zero false alarms in normal traffic.

## 1. Introduction

Road traffic accidents remain one of the leading causes of death worldwide, with 1.19 million fatalities reported annually [1]. A significant proportion involve vulnerable road users—pedestrians, cyclists, motorcyclists—who are frequently occluded from the driver’s view by other vehicles, vegetation, or infrastructure. Cooperative perception through vehicle-to-vehicle (V2V) communication has long been identified as a countermeasure: if one vehicle detects a hazard that another cannot see, sharing this information can prevent dangerous situations [2].

Three research threads have converged around this problem. First, cooperative perception systems have demonstrated effective multi-agent feature fusion for 3D detection, but rely on LiDAR and do not produce explainable risk assessments. Second, single-vehicle LLM-based driving systems have shown that large language models can reason about driving scenes, but operate from a single ego perspective without V2V cooperation. Third, recent frameworks combining LLMs with V2V perception are evaluated exclusively on simulated data with expensive multi-sensor configurations. These three directions are reviewed in detail in Section 2.

Operating on real-world dashcam data introduces challenges absent from simulated environments: asynchronous camera streams with no shared clock, GPS updates limited to 1 Hz (leaving 30 consecutive frames with identical coordinates), variable lighting and weather, and the absence of depth sensors. Furthermore, two vehicles traveling the same road do not observe the same scene simultaneously—one passes through a location first, and the other follows seconds later. Reconstructing this spatial–temporal relationship from monocular video is a prerequisite for cooperative reasoning in this context.

This paper addresses a gap that none of the above systems fill: *cooperative V2V hazard detection on real-world monocular dashcam data*. CoSafe, a five-layer pipeline, is presented and extends a previously validated image-based vehicle positioning algorithm with four new layers: object detection, structured state abstraction, cooperative reasoning, and deterministic safety validation. The LLM serves as a replaceable reasoning layer within this framework. The pipeline is validated on an occluded pedestrian scenario where the leading vehicle detects a pedestrian crossing the road that the following vehicle’s on-board detector cannot reliably perceive in time, due to distance and partial occlusion by the leading vehicle and roadside vegetation. The main contributions of this paper are as follows:

1. A five-layer cooperative perception pipeline operating on asynchronous real-world monocular dashcam streams from multiple vehicles.
2. A compact state abstraction schema encoding kinematic, perceptual, and contextual information with an explicit perception asymmetry signal.
3. A deterministic rule-based safety validation layer providing fail-safe overrides in safety-critical scenarios.
4. The Cooperation Gain metric, quantifying the proportion of cases in which hazard detection depends on V2V information sharing.

The remainder of this paper is organized as follows. Section 2 reviews related work. Section 3 formalizes the problem. Section 4 presents the five-layer architecture. Section 5 describes implementation details. Section 6 presents the experimental evaluation. Section 7 discusses strengths, limitations, and future directions, and Section 8 concludes the paper.

## 2. Related Work

This section reviews three relevant research directions: single-vehicle LLM-based driving systems, cooperative V2V perception frameworks, and image-based vehicle positioning. The section concludes with a comparative summary positioning CoSafe within the existing literature.

### 2.1. Single-Vehicle LLM-Based Driving

The integration of LLMs into autonomous driving has accelerated since 2024. DriveMLM [3] aligns a Multimodal Large Language Model (MLLM) with behavioral planning states for the Apollo/Autopilot system, achieving driving score 76.1 on CARLA Town05 Long, but requires four cameras and LiDAR in simulation. Agent-Driver [4] introduces an LLM cognitive agent with a tool library, cognitive memory, and a reasoning engine performing Chain-of-Thought planning with self-reflection, reducing collisions by over 30% on nuScenes. DriveLM [5] formulates driving as graph visual question answering. DiLu [6] demonstrates knowledge-driven decision-making with reasoning, reflection, and memory modules. Reason2Drive [7] introduces a 600K video-text benchmark with annotated perception–prediction–reasoning chains. LeAD [8] combines a high-frequency end-to-end policy with a redundant LLM module. Nazar et al. [9] propose an agentic framework for distracted driving, achieving 85.7% correctness with 1.74 s latency.

Most recently, NVIDIA’s Alpamayo-R1 [10] (announced at CES 2026) represents the industry’s convergence toward reasoning-based autonomy: a 10.5B-parameter Vision–Language–Action model that pairs a Cosmos-Reason backbone with a diffusion trajectory decoder, producing both trajectory predictions and Chain-of-Causation reasoning traces. However, Alpamayo is single-ego and does not ingest features from neighboring vehicles.

These systems share two common constraints: (a) reliance on a single vehicle’s perspective, and (b) evaluation on simulated or curated datasets rather than uncurated real-world recordings. Furthermore, none includes deterministic safety overrides on LLM outputs, a concern addressed by Wang et al. [11] who propose Model Predictive Control (MPC)-based safety shields for LLM planning.

### 2.2. Cooperative V2V Perception

Traditional cooperative perception focuses on efficient feature sharing for 3D detection. Where2comm [12] introduces spatial confidence maps for communication-efficient collaboration. V2X-ViT [13] applies heterogeneous multi-agent self-attention transformers handling asynchronous sharing and pose errors. These approaches operate on LiDAR data and are task-narrow, producing detection outputs without risk assessments or explainable reasoning.

More recently, LLM-based cooperative frameworks have emerged. V2V-LLM [14] (NVIDIA/CMU) is the first to apply multimodal LLMs to cooperative V2V driving, sharing LiDAR perception features into a shared MLLM via the V2V-QA dataset built on V2V4Real [15]. V2V-GoT [16] extends this with a Graph-of-Thoughts reasoning scaffold adding occlusion-aware perception. Talking Vehicles [17] explores V2V communication through natural language. AgentsCoDriver [18] proposes a multi-vehicle LLM with lifelong learning. All of these systems are evaluated on simulated data with LiDAR-derived features; none operates on real monocular dashcam video.

### 2.3. Positioning of This Work

This work builds on the prior image-based vehicle positioning framework. In [19], a decentralized algorithm for relative vehicle positioning was proposed using ORB [20] keypoints with BEBLID [21] descriptors and brute-force matching with Lowe’s ratio test [22], achieving sub-meter positioning accuracy validated against physical measurements, significantly outperforming GPS-only estimation. In [23], a centralized cloud architecture was proposed for the same algorithm. In [24], SegFormer-B4 [25] preprocessing was integrated, increasing average match counts from 250 to 1150 per frame by filtering vegetation and sky. The present work extends this positioning foundation with four new layers for cooperative hazard reasoning.

Table 1 summarizes how the proposed approach compares to existing systems.

The present work addresses the intersection of these three open challenges: operating on real monocular dashcam data for V2V cooperation, requiring no LiDAR infrastructure, and incorporating a deterministic safety override layer alongside LLM reasoning.

## 3. Problem Formulation

This section formalizes the cooperative V2V hazard detection problem as follows.

**Input.** Two asynchronous video streams $V^{A}=\left\{f_{1}^{A},\ldots ,f_{N}^{A}\right\}$ and $V^{B}=\left\{f_{1}^{B},\ldots ,f_{M}^{B}\right\}$ captured by forward-facing monocular cameras on vehicles *A* (leading) and *B* (following), with associated GPS metadata $G^{A},G^{B}$ providing position, speed, and timestamps at 1 Hz.

**Challenges.**

- *Temporal offset:* The two vehicles pass through the same location at different times; vehicle *B* follows vehicle *A* with a variable time delay.
- *Partial observability:* Each vehicle observes only its own forward view; hazards visible to *A* may be occluded from *B* by *A* itself, vegetation, or other obstacles.
- *Asymmetric perception:* The set of detectable objects differs between vehicles due to viewpoint and occlusion differences.
- *Limited sensor fidelity:* Both vehicles use monocular cameras with 1 Hz GPS updates; no depth sensing, no LiDAR, and no sub-frame temporal resolution are available.

**Goal.** Given the two streams, infer a cooperative scene understanding that resolves perceptual asymmetries and produces for each aligned frame pair $\left(f_{i}^{A},f_{j}^{B}\right)$ the following:

1. A risk assessment r∈{LOW,MEDIUM,HIGH,CRITICAL};
2. A scenario classification *s* (e.g., pedestrian, occlusion, normal);
3. An action recommendation *a* (e.g., maintain, prepare_stop);
4. Explainable warnings $w^{A},w^{B}$ for each vehicle.

**Key constraint.** The system must satisfy the *safety monotonicity* property: the final risk assessment after the safety layer must be at least as conservative as any individual rule-triggered escalation, i.e., $r_{\mathrm{final}}\geq r_{\mathrm{safety}}$ for all active safety rules, regardless of LLM output, where risk levels are ordered as LOW<MEDIUM<HIGH<CRITICAL.

## 4. Proposed Architecture

**Figure 1:**
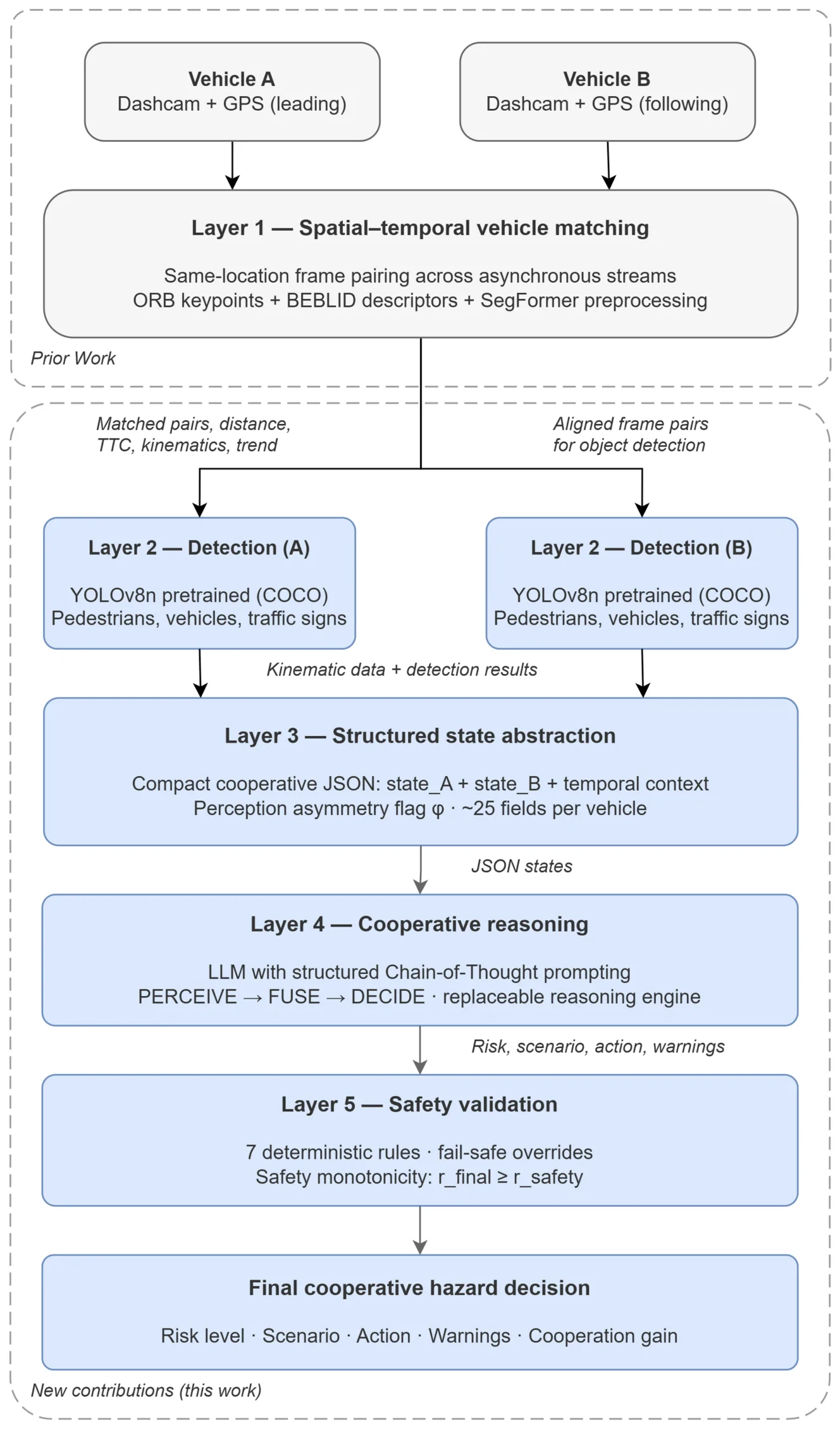
Five-layer cooperative V2V hazard detection pipeline. Layer 1 (spatial–temporal matching) is reused from prior work [19,24]. Layers 2–5 are new contributions. The dashed boxes group the prior-work component (top) and the new contributions of this work (bottom).

CoSafe is organized as a five-layer pipeline (Figure 1), where each layer consumes the output of the previous one. Layer 1 reuses the previously validated positioning algorithm; Layers 2–5 represent the contributions of this work. The pipeline currently operates offline on recorded data, though the modular design is compatible with real-time deployment via edge computing.

### 4.1. Layer 1: Spatial–Temporal Vehicle Matching

In this paper, “V2V” denotes the cooperative use of information from two vehicles; the communication channel itself is not implemented. Cooperation is reconstructed offline from two independently recorded dashcam and GPS streams, which isolates the perception and reasoning problem from the networking problem of delivering that information live. The effects of a real communication channel (latency, packet loss, bandwidth) are discussed in Section 7.

This layer solves the frame correspondence problem: for each frame $f_{i}^{A}$, find the frame $f_{j}^{B}$ that depicts the *same geographic location*, not the same time instant. This distinction is fundamental: vehicle *A* passes first, and vehicle *B* follows seconds later through the same location. Reconstructing this spatial–temporal relationship is what enables cooperative perception. While spatial matching provides the inter-vehicle distance, perception is compared on temporally aligned frames: the frame from vehicle *A* captured at the same instant as the current frame from vehicle *B*. This ensures that perception asymmetry reflects what both vehicles observe simultaneously, rather than at different times.

The matching pipeline follows [19]: ORB [20] keypoints are detected per frame, BEBLID [21] binary descriptors are computed, and a BFMatcher with Hamming distance and Lowe’s ratio test [22] is applied, selecting $j^{*}=\arg\max_{j}\left|M\left(f_{i}^{A},f_{j}^{B}\right)\right|$. SegFormer-B4 [25] preprocessing (fine-tuned on CityScapes [26]) filters vegetation and sky to improve match quality [24]. Inter-vehicle distance is computed from timestamp differences and GPS speeds as described in [19].

Cooperation assumes that the two vehicles traverse the same location within a bounded time window. The spatial match is accepted only when the feature-matching quality between the two frames exceeds a threshold, which implicitly bounds the admissible time gap: as the interval between the two passages grows, the scene changes (different traffic, lighting, and moving objects) and the visual match quality degrades until the pair is rejected. In the evaluated scenario the two vehicles pass each matched location within a few seconds of each other, well within this window. For very large time gaps the approach is not applicable, since a hazard observed by the leading vehicle may no longer be present when the following vehicle arrives; in that regime the shared observation is stale and the cooperative signal is correctly suppressed by the low match quality rather than propagated as a false hazard. Characterizing the exact usable time window across diverse conditions is left to future work.

This layer additionally computes kinematic metrics novel to the present work:

**Time-to-Collision (TTC):**

(1) $$\mathrm{TTC}=\left\{\begin{array}{cc} d/v_{\mathrm{close}},\; & v_{\mathrm{close}}>0,\\ \infty ,\; & v_{\mathrm{close}}\leq 0, \end{array}\right.$$

where *d* is the estimated inter-vehicle distance and $v_{\mathrm{close}}$ is the closing speed derived from frame-to-frame distance changes.

**Distance trend slope:** The slope β of a linear regression d(t)=α+βt fitted to the last *W* consecutive distance measurements, classifying dynamics as closing (β<−ϵ), stable (|β|≤ϵ), or separating (β>ϵ). A negative slope indicates that the inter-vehicle distance is decreasing over time, suggesting the following vehicle is approaching the leading one.

**Kinematic risk score:**

(2) $$r_{\mathrm{kin}}=\min\;\left(1,\;\frac{v_{\mathrm{close}}}{10}+\frac{1_{\left\{\mathrm{TTC}<10\right\}}}{\mathrm{TTC}}+\frac{\left|\beta \right|}{2}\right),\;r_{\mathrm{kin}}\in \left[0,1\right].$$

The kinematic risk score combines three normalized components: the closing speed relative to a reference value, the inverse of TTC when it falls below a safety horizon, and the magnitude of the distance trend slope. Higher values of $r_{\mathrm{kin}}$ indicate more dangerous kinematic configurations. The score is clamped to [0,1] to provide a uniform input to the safety layer.

**Match confidence and quality:** The ratio of good matches to the local maximum, with a *z*-score relative to running statistics. Quality is labeled normal if confidence exceeds a predefined threshold, low otherwise.

### 4.2. Layer 2: Semantic Object Detection

YOLOv8n [27] (Ultralytics, COCO weights, zero fine-tuning) is applied to the temporally aligned frame pair identified in Layer 1, detecting pedestrians, vehicles, bicycles, traffic lights, and stop signs. YOLOv8 was selected as the most widely adopted real-time object detection architecture, with the nano variant chosen to enable CPU-only inference consistent with the low-cost hardware constraint of this work. For each detection, a spatial position is classified on a 3×3 grid (left/center/right × far/middle/near) and a relative size label is assigned (small/medium/large). Potential occluders (trucks, buses on lateral positions) are flagged explicitly.

The pretrained model is used without fine-tuning to evaluate whether off-the-shelf detection weights transfer to uncurated real-world dashcam footage captured under uncontrolled conditions.

### 4.3. Layer 3: Structured State Abstraction

This layer is the central methodological contribution. It transforms heterogeneous outputs from Layers 1–2 into a compact structured representation designed for automated reasoning. The design follows two principles: *minimality* (only safety-relevant fields, reducing token count and hallucination risk) and *explicitness* (pre-computed derived quantities rather than raw data).

Each state pair $\left(s^{A},s^{B},c_{t}\right)$ consists of the following:

- **Per-vehicle state** (∼25 fields): kinematic data (*d*, *v*, TTC, $v_{\mathrm{close}}$, trend, $r_{\mathrm{kin}}$), matching quality (confidence, *z*-score, quality label), scene context (lane count, lateral occupancy), and detection summaries (pedestrian visible/count, vehicle counts, traffic lights, occlusions).
- **Perception asymmetry flag: **$\phi =1_{\left\{P^{A}>0\wedge P^{B}=0\right\}\vee \left\{P^{A}=0\wedge P^{B}>0\right\}}$, where $P^{V}$ is the pedestrian count for vehicle *V* above a confidence threshold. This flag encodes the core value of cooperative perception.
- **Temporal context **$c_{t}$: sliding-window statistics over recent pairs—dominant trend, persistent closing flag, minimum TTC, average match confidence, distance delta.

In the current implementation, ϕ is defined over pedestrian detections; extension to other object classes is straightforward but not evaluated in this work.

### 4.4. Layer 4: Cooperative Reasoning

A cloud-hosted LLM receives $\left(s^{A},s^{B},c_{t}\right)$ and produces a structured decision through three-phase Chain-of-Thought prompting the following:

1. **PERCEIVE:** Analyze each vehicle’s observations independently; identify objects, occlusions, kinematic context; use temporal context to distinguish noise from persistent patterns.
2. **FUSE:** Combine perspectives; identify perception asymmetries, the core value of cooperative sensing, and assess what V2V adds.
3. **DECIDE:** Output structured JSON: risk level, scenario type, action, per-vehicle warnings, explanation referencing input fields, and self-assessed confidence.

The prompt includes explicit grounding rules preventing common failure modes: (a) if any vehicle has pedestrian_visible = true, risk cannot be LOW; (b) if visual descriptions conflict with structured detections, trust structured fields; (c) if match quality is low, reduce confidence. These rules were iteratively refined based on observed LLM behavior during development. The complete prompt is provided in Appendix A. The risk level is assigned in two stages. First, the reasoning layer proposes a level (LOW, MEDIUM, HIGH, or CRITICAL) from the fused state, guided by explicit criteria in the prompt: the presence and position of a pedestrian, the time-to-collision, the kinematic risk score, and whether a perception asymmetry is present. A pedestrian visible to either vehicle forces at least MEDIUM; a pedestrian at close range with a confirmed asymmetry forces at least HIGH. Second, the safety layer can only escalate this level through the deterministic rules described in Section 4.5, never reduce it. The final level is therefore the more conservative of the reasoning output and the safety rules. LOW is reserved for situations with no pedestrian, no critical motion cue, and no corridor restriction; CRITICAL corresponds to imminent collision conditions (very low time-to-collision or critical closing distance). This separation keeps the qualitative reasoning of the language model subordinate to hard, auditable safety thresholds.

The system supports both cloud-based and local LLM providers, with output constrained to JSON format and inference parameters listed in Table 2. The system was evaluated with models hosted via Groq. Because the LLM is a replaceable component, the specific model was updated during development as provider availability changed; the models used and the impact of model selection on latency are reported in Section 5 and Section 6.3.

### 4.5. Layer 5: Safety Validation

The safety layer implements the principle that the reasoning engine should *enhance* safety decisions but never be the sole arbiter in safety-critical scenarios. LLMs are inherently probabilistic: they can hallucinate facts not present in the input, produce inconsistent risk assessments across similar inputs, or fail silently on out-of-distribution scenarios [11]. In automotive applications, such failures can have irreversible consequences. The safety layer therefore provides a deterministic lower bound on the system’s response to critical situations, independent of LLM output quality.

Seven rules are defined, each with explicit trigger conditions and override actions:

1. **Critical distance:** (*d* below critical threshold) and (trend = closing) ⇒ CRITICAL/emergency_brake.
2. **High closing speed:** ($v_{\mathrm{close}}$ above speed threshold) and (action = maintain) ⇒ escalate to slow_down.
3. **Critical TTC:** (TTC below critical threshold) ⇒ CRITICAL; (TTC below warning threshold) and (risk < HIGH) ⇒ escalate.
4. **Low match confidence:** (confidence below minimum threshold) ⇒ flag uncertain; escalate maintain to maintain_caution.
5. **Occlusion escalation:** (occlusion present) and (risk = LOW) ⇒ escalate to MEDIUM.
6. **Pedestrian proximity:** (ϕ=1) and (*d* below proximity threshold) ⇒ HIGH/prepare_stop/pedestrian. This rule directly encodes the cooperative perception benefit.
7. **Critical kinematic risk:** ($r_{\mathrm{kin}}$ above critical threshold) ⇒ CRITICAL; ($r_{\mathrm{kin}}$ above warning threshold) and (risk < HIGH) ⇒ escalate.

Rules are evaluated sequentially and multiple rules may fire on the same decision. When this occurs, the most conservative outcome is retained, satisfying the safety monotonicity property defined in Section 3. Each override is logged with the triggering rule, original LLM values, and corrected values, providing full auditability. The output includes a source field distinguishing LLM-originated decisions from safety-overridden ones.

Rule 6 is architecturally significant: it is the only rule that reasons across both vehicles simultaneously. If vehicle *A* detects a pedestrian via YOLO and vehicle *B* does not (i.e., ϕ=1), and the inter-vehicle distance is below the safety threshold, the system escalates the risk to HIGH and sets action to prepare_stop regardless of the LLM’s assessment. This ensures that the core cooperative perception signal—one vehicle seeing what the other cannot—always reaches the final decision.

The safety layer is constructed so that it can only raise the risk level, never lower it. Let the risk levels be totally ordered as LOW<MEDIUM<HIGH<CRITICAL, and let $r_{\mathrm{LLM}}$ be the level proposed by the reasoning layer. Each safety rule *k* is a guarded escalation: when its precondition holds it proposes a level $r_{k}$ and the current level is updated to $\max(r_{\mathrm{current}},r_{k})$; when its precondition does not hold it leaves the level unchanged. Since every rule either raises the level to its associated value or does nothing, and the maximum operator is commutative and associative, the order in which the rules are evaluated does not affect the outcome, and the final level satisfies

(3) $$r_{\mathrm{final}}=\max\;\left(r_{\mathrm{LLM}},\;\max_{k\in T}r_{k}\right)\;\geq \;r_{\mathrm{LLM}},$$

where T is the set of rules whose preconditions are satisfied. Consequently $r_{\mathrm{final}}\geq r_{\mathrm{safety}}$ for every rule that fires, and no combination of simultaneously triggered rules can downgrade the risk below any individual rule’s requirement. When several rules fire together, the most conservative outcome prevails by construction. The one exception is the fallback path: if the reasoning layer returns malformed or missing output, the layer discards it entirely and issues a fixed cautious default (maintain_caution), treated as a floor rather than an escalation.

The validation procedure is as follows:

1. If the LLM decision is malformed or missing, discard it and return a fixed cautious default (maintain_caution) with an uncertainty flag.
2. Otherwise, initialize the risk level *r* and action *a* from the LLM output.
3. For each rule *k* in the rule set: if its precondition holds on the fused states, update $r\leftarrow \max(r,r_{k})$, escalate the action accordingly, and log the override.
4. Return the final risk level, action, and override log.

## 5. Implementation Details

This section describes the hardware setup, software components, configuration parameters, and evaluation metrics used in the CoSafe pipeline.

### 5.1. Hardware and Data Acquisition

Data was acquired by the authors using two DDPAI MOLA N3 dashboard cameras mounted on two vehicles driving urban streets under real traffic conditions. Each camera records 2K resolution video at 30 fps with integrated GPS providing position and speed in NMEA format ($GPRMC/$GPGGA sentences) at 1 Hz. Since the GPS updates once per second, 30 consecutive frames share identical coordinates; sub-second positioning is recovered through frame interpolation using the method described in [19]. Although both cameras are the same model and resolution, a hardware revision introduced minor differences in GPS coordinate encoding within the NMEA data stream, which are handled transparently by the parsing layer.

All processing is performed offline on a standard laptop without GPU acceleration. The pipeline does not require specialized hardware beyond the two dashboard cameras themselves.

### 5.2. Software Stack and Model Selection

The pipeline is implemented in Python 3.14 using OpenCV 4.10 with ORB and BEBLID (cv.xfeatures2d) for frame matching, SegFormer-B4 via HuggingFace transformers for semantic segmentation preprocessing, YOLOv8n via Ultralytics (∼6 MB model) for object detection, and Groq API for LLM reasoning. A central orchestrator script invokes each layer sequentially, with all inter-layer communication through JSON files.

YOLOv8n was selected as the smallest variant in the YOLOv8 family, prioritizing inference speed on CPU over detection accuracy. No fine-tuning was performed to test whether off-the-shelf COCO-trained weights generalize to real dashcam conditions.

For LLM reasoning, Groq was selected for its free-tier API access and low-latency inference on large models. An early experiment with a small model (Qwen 2.5 1.5B via Ollama) failed to identify perception asymmetries in nearly all test cases, motivating the use of larger hosted models. Because model availability on the provider changed during the study, the initial 30-pair subset used meta-llama/llama-4-scout-17b-16e-instruct, while the extended 60-pair and 21-pair negative sets used qwen/qwen3.8-27b; the effect on latency is reported in Section 6.3. Ollama remains available as a fully offline alternative for environments without internet access.

### 5.3. Configuration Parameters

Table 2 lists the threshold values used across all pipeline layers. These values were calibrated empirically during development on the test scenario described in Section 6.

### 5.4. Cooperation Gain

The central evaluation metric of this work measures the proportion of frame pairs in which a hazard is present but cannot be reliably perceived by the following vehicle on its own, so that its detection depends on cooperation:

(4) $$\mathrm{CG}=\frac{\left|\{p\in P:\mathrm{detected}\left(p\right)\wedge \neg {\mathrm{visible}}_{\mathrm{ego}}\left(p\right)\}\right|}{\left|P\right|},$$

where *P* is the set of evaluated frame pairs, detected(p) indicates that the system escalates the pair to a hazard (HIGH or CRITICAL), and ${\mathrm{visible}}_{\mathrm{ego}}\left(p\right)$ indicates that the following vehicle could itself perceive the pedestrian in that pair. Crucially, ${\mathrm{visible}}_{\mathrm{ego}}$ is determined from an independent human annotation of the following vehicle’s view (Section 6.2), not from the system’s own detector, so the metric does not reduce to a statement about the pipeline’s internal consistency. A pair counts toward the Cooperation Gain only when the pedestrian was not clearly visible to the following vehicle yet the system still flagged the hazard using the leading vehicle’s information.

Reported this way, the Cooperation Gain for the evaluated scenario is 0.533: in 32 of the 60 pairs the pedestrian was below the following vehicle’s clear visibility (annotated no or unclear) and the hazard was available only through cooperation, while in the remaining 28 pairs the pedestrian was already clearly visible to the following vehicle. Measuring against an independent reference rather than the system’s own detections yields a lower but more meaningful value.

Additional metrics include the safety override rate (proportion of decisions modified by Layer 5), LLM inference latency, and the decision distribution across risk levels.

## 6. Experimental Evaluation

This section presents the test scenario, pipeline results across 60 frame pairs, and a detailed end-to-end trace of a representative example.

### 6.1. Test Scenario

CoSafe was evaluated on an occluded pedestrian scenario recorded on urban streets. Two vehicles equipped with dashboard cameras traveled the same road segment, with Vehicle A (leading) approximately 9–10 m ahead of Vehicle B (following). Both vehicles traveled at approximately 22–23 km/h with stable inter-vehicle distance (trend = stable, TTC = *∞*).

As Vehicle A exited a narrow street bordered by dense vegetation into an open area, a pedestrian began crossing the road ahead. Vehicle A had a clear, unobstructed view of the pedestrian. For Vehicle B, following behind, the pedestrian was initially fully occluded and then only progressively and faintly visible due to distance and partial occlusion by the leading vehicle and roadside vegetation, remaining below the threshold of reliable automated detection for an extended interval. This scenario represents the core use case for cooperative perception: a hazard reliably perceived by one vehicle while the other’s local perception fails to detect it in time.

**Figure 2:**
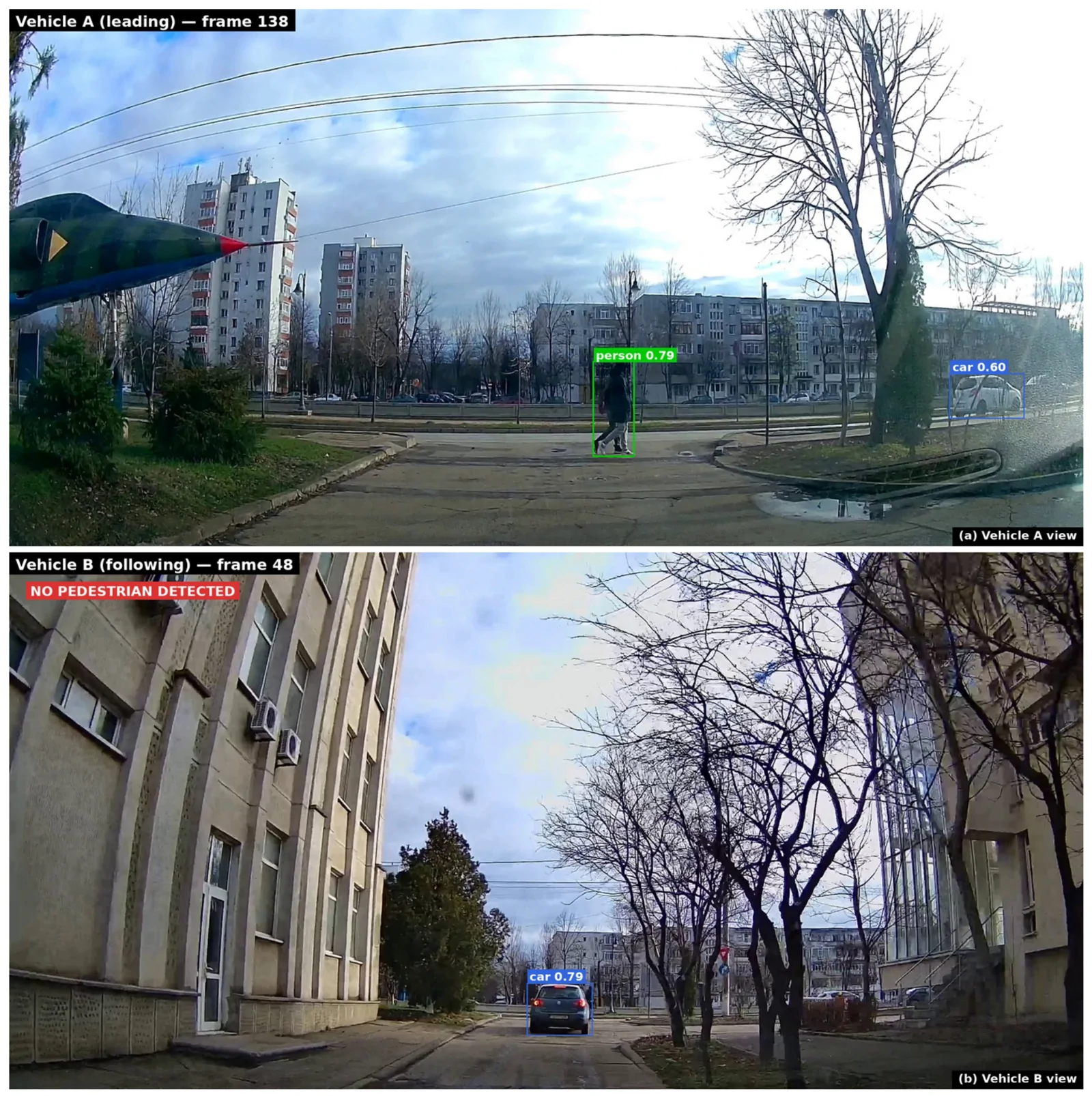
Perception asymmetry in the occluded pedestrian scenario. (**a**) Vehicle A detects a pedestrian (confidence 0.79) crossing ahead. (**b**) Vehicle B’s on-board detector registers only a vehicle; the pedestrian is below its reliable-detection threshold due to distance and partial occlusion by vegetation and the leading vehicle. This asymmetry triggers the cooperative perception signal (ϕ=1).

The sequence spans the full occlusion-to-visibility transition, from the pedestrian’s first appearance in the leading vehicle’s view until the following vehicle’s detector first registers the pedestrian, allowing both the cooperative benefit and its temporal extent to be measured. Figure 2 illustrates the perception asymmetry for a representative frame pair from within the occlusion window.  

### 6.2. Human Ground-Truth Annotation

Evaluating the pipeline against its own detection outputs alone would measure self-consistency rather than correctness. To provide a reference independent of the system’s detector, one of the authors manually annotated the visibility of the pedestrian in the following vehicle’s view across the full sequence, through frame-by-frame visual inspection, using a three-level scale: no (nothing distinguishable from the background), unclear (a possible person, but ambiguous due to distance or partial occlusion), and clear (an unmistakable human form). The leading vehicle’s view was annotated clear throughout, consistent with its detector.

Table 3 summarizes the annotation. The annotation shows a clear transition. The pedestrian is not distinguishable in the early frames, becomes progressively but ambiguously visible through the middle of the sequence, and reaches a confident human-visible threshold well before the following vehicle’s automated detector first registers it, a gap quantified in Section 6.3.

This annotation clarifies the nature of the scenario. It is not a case of total physical occlusion throughout; rather, the pedestrian is visible in principle but remains below the threshold of reliable automated detection in the following vehicle’s view for an extended interval, due to distance and partial occlusion by the leading vehicle and roadside vegetation. Cooperation supplies the following vehicle with the leading vehicle’s confident detection during precisely this window.

### 6.3. Pipeline Results

The scenario was evaluated over 60 consecutive temporally aligned frame pairs, spanning from the pedestrian’s first appearance in the leading vehicle’s view, through the full occlusion window, until the following vehicle’s on-board detector first registered the pedestrian. Table 4 summarizes the pipeline output.

The leading vehicle’s detector registers the pedestrian in all 60 frames (confidence 0.46–0.86). The following vehicle’s detector registers it in only 3 of 60 frames, and only in the late part of the sequence when the pedestrian has moved into an unobstructed, close position. This asymmetry (ϕ=1 in 57 of 60 pairs) is the condition the cooperative layer is designed to exploit.

The LLM assessed the risk as HIGH in 44 of 60 pairs and MEDIUM in the remaining 16. The safety layer escalated the 16 MEDIUM cases to HIGH through the pedestrian-proximity rule, and additionally raised a low-match-confidence flag on 60 pairs (76 overrides in total), yielding a unanimous final assessment of HIGH/pedestrian/prepare_stop across all 60 pairs.

#### 6.3.1. Detector Performance Against Human Ground Truth

To assess the following vehicle’s detector against an independent reference, its YOLO detections were compared with the human annotation of clear pedestrian visibility (Section 6.2). The detector reaches a precision of 1.00 but a recall of only 0.107: it fires correctly when it fires, but misses the pedestrian in 25 of the 28 frames a human annotator judged clearly visible. This large detector–human gap is the core motivation for cooperation: the following vehicle’s own perception is, on this data, an unreliable basis for hazard detection, whereas the leading vehicle supplies a confident detection throughout.

#### 6.3.2. Measured Cooperation Gain

Measured against the human ground truth rather than the system’s own output, the Cooperation Gain is 0.533: in 32 of 60 pairs the following vehicle could not itself perceive the pedestrian clearly (annotated no or unclear) and therefore depended on the cooperative channel; in the remaining 28 pairs the pedestrian was already clearly visible to the following vehicle. Reporting against an independent reference yields a lower value than a figure computed from the system’s own detections, and reflects how often cooperation adds information beyond what the following vehicle already perceives.

#### 6.3.3. Latency

Reasoning latency depends on the language model used. The initial experiments used meta-llama/llama-4-scout-17b-16e-instruct at a mean of 3.47 s per decision; this model was subsequently deprecated by the inference provider, and the extended evaluation used qwen/qwen3.8-27b at 20.75 ± 3.51 s (median 21.14 s). Since the LLM is a replaceable layer, latency can be traded against reasoning quality through model selection; the pipeline itself is agnostic to the specific model.

### 6.4. Ablation Study

To isolate the contribution of each pipeline component, five configurations were evaluated on an extended set of 60 temporally aligned frame pairs spanning the full occlusion sequence. In b_only, only the following vehicle’s perception is used; in a_only, only the leading vehicle’s. rules_only skips the LLM and applies the safety rules directly to the fused state; llm_only runs the LLM but disables the safety layer; and full is the complete pipeline.

Table 5 reports the results. The following vehicle on its own detects the hazard in only 3 of 60 pairs—the three frames where the pedestrian becomes visible to its own detector. In every other frame it registers nothing. This is the clearest evidence that the hazard information comes from cooperation rather than from B’s local sensor. Once the cooperative state is available, the deterministic rules already handle the scenario on their own (rules_only reaches 1.00). The LLM alone is weaker here: it marks 16 pairs as MEDIUM instead of HIGH, and the safety layer is what raises them back to HIGH in the full system.

The role of the LLM in this ablation deserves careful interpretation. On this specific scenario the deterministic rules match the full pipeline, because the pedestrian-proximity rule was designed to cover exactly this type of hazard. This does not make the LLM redundant. The deterministic rules are hand-crafted for known hazard categories; they fire correctly here only because such a rule was written in advance. The LLM reasoning layer, by contrast, produces a risk assessment and a natural-language justification for situations that no predefined rule anticipates—novel hazard types, unusual combinations of cues, or contexts outside the rule set. The ablation therefore shows two complementary mechanisms: a deterministic layer that guarantees correct behavior on covered cases, and a reasoning layer that extends coverage to unanticipated ones. The safety layer additionally acts as a backstop, recovering the 16 cases the LLM alone under-rated. What the ablation establishes unambiguously is that cooperation—not the reasoning mechanism—is the decisive factor for detection: without it, the following vehicle detects almost nothing (0.05).

### 6.5. Negative Scenario Test

A cooperative system must not only detect genuine hazards but also remain silent when none are present. To assess specificity, the pipeline was evaluated on a separate normal-traffic recording of 21 frame pairs containing no pedestrian: a one-way street with parked vehicles on both sides and a car braking toward a STOP sign. The absence of any pedestrian was confirmed in both vehicle views prior to processing.

Table 6 reports the outcome. No configuration produced a single false positive: no pair was escalated to HIGH or CRITICAL risk. Two pairs received a MEDIUM assessment, but these are not false alarms. Both were classified as constrained_road—a narrow corridor with parked vehicles on both sides—with low kinematic risk (score 0.17) and no collision threat (TTC > 100 s). The reasoning layer explicitly flagged the narrow corridor while noting the absence of immediate danger, which is the intended cautious behavior rather than a fabricated hazard. No safety-layer override was triggered on the negative set.

The negative test confirms that the system does not signal hazards indiscriminately. It distinguishes a genuine pedestrian hazard from a merely constrained but safe context, and does not manufacture hazards where none exist.

Combining the positive and negative sets (81 frame pairs in total) gives a system-level view: the full pipeline reaches 60 true positives, 21 true negatives, and zero false positives or false negatives against the hazard-present ground truth, for a precision, recall, and $F_{1}$ of 1.00. The llm_only configuration reaches a recall of 0.733 at precision 1.00, the gap that the safety layer closes.

A concrete instance of this appears on the negative set: the rules-only configuration labels all 21 pairs LOW, whereas the LLM (and the full pipeline) flags two pairs as constrained_road MEDIUM, correctly identifying a narrow corridor with parked vehicles on both sides. This contextual nuance came from the reasoning layer, not from the fixed rules, illustrating the LLM’s contribution beyond the covered hazard categories.

### 6.6. Advance Warning Time

The central benefit of cooperative perception is temporal: the leading vehicle can perceive a hazard before the following vehicle has any means of detecting it. To quantify this, the detection timelines of both vehicles were compared across the extended sequence. The leading vehicle detects the pedestrian from the first available frame onward. The following vehicle’s on-board detector registers the pedestrian only much later, once the pedestrian has moved into an unobstructed, sufficiently close position.

The leading vehicle detects the pedestrian at least 52 frames before the following vehicle’s detector does, corresponding to approximately 1.73 s at the recording rate, or about 12 m of travel at the recorded speed of roughly 25 km/h. This is a lower bound, since the leading vehicle already detected the pedestrian in the first frame of the available window.

This margin combines two distinct effects, which the human annotation lets us separate. For the first part of the window the pedestrian is genuinely below human visibility in the following vehicle’s view (annotated no or unclear), a true perceptual occlusion of roughly 1.07 s. For the remaining part, a human annotator could already see the pedestrian but the on-board YOLOv8n detector still failed to register it, contributing about 0.66 s. The former is the intrinsic cooperative benefit; the latter reflects a limitation of the specific detector, which a stronger on-board model would reduce. Reporting both makes clear that the cooperative advantage does not rest solely on a weak detector.

A caveat concerns the reasoning latency. The advance-warning window (1.73 s) is shorter than the measured LLM latency (Section 6.3), so the language model alone could not deliver a warning within the window. In the proposed architecture this is handled by the separation of layers: the deterministic safety layer operates on the structured state almost instantly and produces the time-critical HIGH/prepare_stop decision, while the LLM supplies the human-readable explanation that need not be on the critical path. The safety-critical output therefore fits within the window even though the full reasoning does not.

### 6.7. Sensitivity Analysis

A recurring concern with rule-based systems is that their thresholds are tuned to the evaluation scenario and would fail elsewhere. To examine this, the two most influential parameters—the pedestrian-proximity distance and the frame-matching confidence threshold—were swept across a range of values on the combined positive and negative sets, holding all other parameters fixed.

Table 7 and Table 8 report the sweeps. The pedestrian-proximity threshold shows a stable plateau between 12 m and 20 m, over which detection remains at 1.00 and the false-alarm rate at zero. Performance degrades only at a very strict 8 m setting (0.83), where some pedestrian–vehicle distances fall outside the rule. The calibrated value of 15 m sits in the middle of this stable region rather than on an edge, indicating that the result does not depend on a finely tuned threshold. The match-confidence threshold has no effect on the final decision across the tested range: it contributes an uncertainty flag rather than altering the risk outcome.

Because the framing of the scenario depends on the following vehicle’s detector missing the pedestrian, the YOLO confidence threshold itself was also swept (Table 9). The advance-warning time is unchanged across all three settings (1.73 s): the position of the following vehicle’s first detection in the sequence does not move, only the total number of detected frames. Detection rate remains at 1.00 for thresholds 0.25 and 0.35, and drops marginally to 0.983 at 0.50. That single lost pair (a175) is caused by the leading vehicle’s own detection confidence falling below 0.50, not by a failure of the cooperative mechanism. The result confirms that the reported behavior is robust to the detection threshold over a reasonable range.

These sweeps were performed on a limited set of two recordings, so the observed stability holds for this data rather than constituting a general guarantee. Nonetheless, it shows that the reported results are not an artefact of a single fragile threshold setting.

### 6.8. End-to-End Pipeline Trace

**Figure 3:**
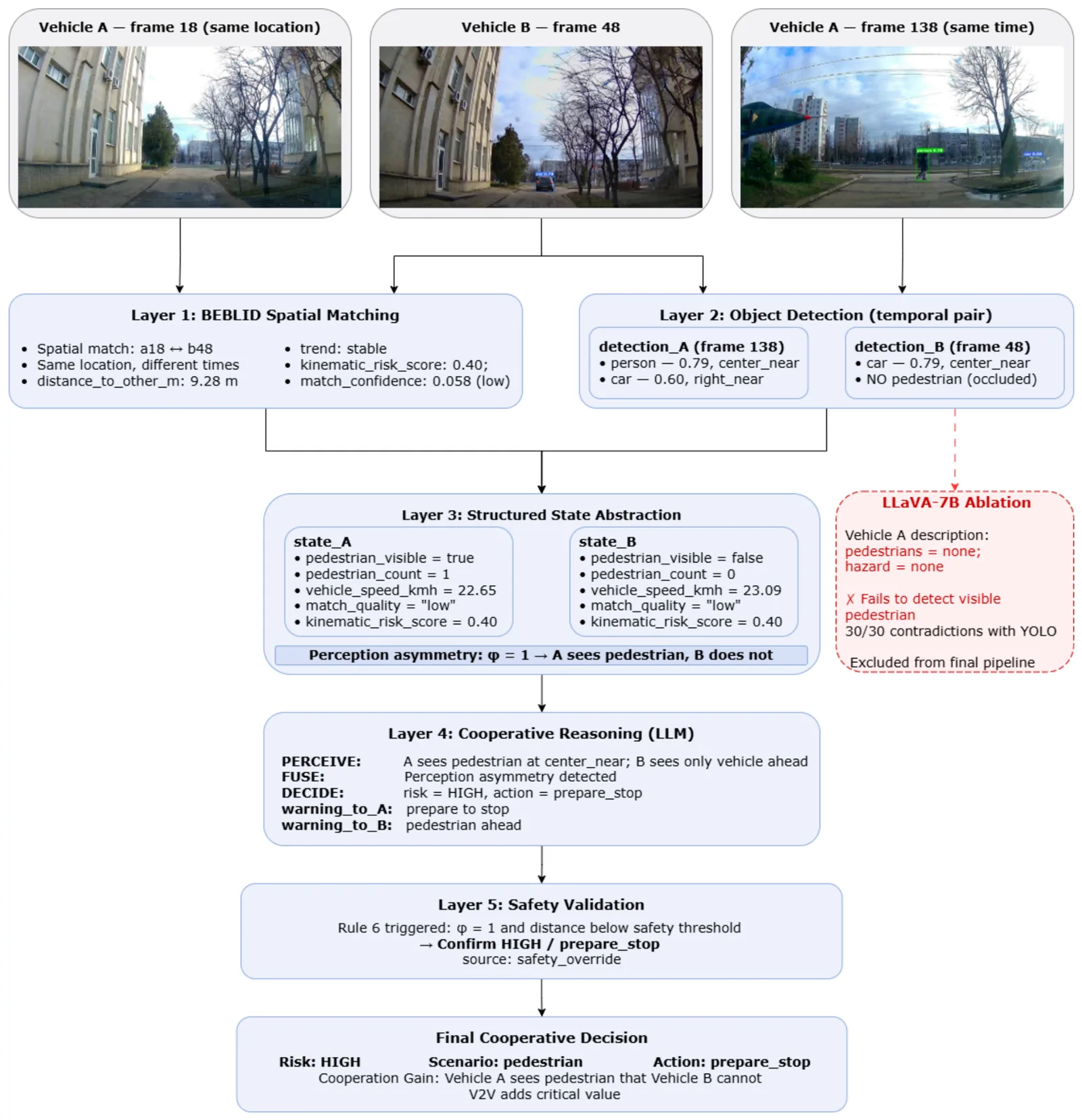
End-to-end pipeline trace for the spatial pair $a_{18}$/$b_{48}$ (distance) and temporal perception pair $a_{138}$/$b_{48}$. Vehicle A detects a pedestrian that Vehicle B’s on-board detector does not register. The LLaVA-7B ablation (right) incorrectly reports no pedestrians, confirming its exclusion from the pipeline. Both the cooperative reasoning layer and safety validation converge on HIGH/prepare_stop. Arrows indicate the flow of data through the pipeline; the dashed red box marks the LLaVA-7B ablation branch, excluded from the final pipeline.

Figure 3 presents the complete pipeline trace for a representative frame pair (a18/b48 spatial, a138/b48 temporal), showing the data flow from raw frames through all five layers to the final cooperative decision. The figure also includes the LLaVA-7B ablation result, which incorrectly reported no pedestrians in the same frame where YOLOv8 detected one with high confidence.

## 7. Discussion

This section discusses the strengths of the proposed architecture in the context of the experimental results, and identifies current limitations and future directions.

### 7.1. Strengths of the Proposed Architecture

The primary strength of this work is its unique positioning within the literature: no prior work demonstrates cooperative V2V hazard reasoning on real monocular dashcam data without requiring LiDAR or dedicated V2V communication hardware. The spatial–temporal matching—where two vehicles traverse the same location at different times and reconstruct their relationship through visual feature correspondence—is architecturally distinct from all existing V2V frameworks that assume synchronized multi-sensor platforms.

The five-layer separation (matching → perception → abstraction → reasoning → safety) provides a clean modular architecture where any layer can be upgraded independently. In particular, the LLM in Layer 4 is a replaceable component—the framework is not tied to any specific model or provider.

The safety layer addresses a known gap in LLM driving research. While Wang et al. [11] propose MPC-based safety shields and the broader community is developing formal guardrails, most existing LLM driving systems (DriveMLM, Agent-Driver, V2V-LLM, Alpamayo) do not include any deterministic override mechanism. The proposed rule-based approach is simpler than formal verification but directly addresses the most critical failure modes in cooperative perception scenarios. A similar separation between probabilistic LLM reasoning and deterministic safety validation has recently been adopted outside the driving domain, e.g., for LLM-driven control of RF measurement instrumentation in smart-city monitoring [28], suggesting that rule-based validation layers are emerging as a general design pattern for LLM-in-the-loop cyber-physical systems.

The temporal context (sliding window with trend analysis, TTC evolution, distance slope) elevates the system beyond a frame-level approach and provides the reasoning layer with trajectory-level awareness.

The experimental evaluation confirms the architectural design. On the extended 60-pair set, the safety layer escalated the 16 cases the LLM under-rated as MEDIUM back to HIGH, demonstrating that the deterministic override mechanism fulfills its intended role. Measured against manual human annotation independent of the system’s detector, the Cooperation Gain is 0.533, and the following vehicle’s own detector reaches a recall of only 0.107 against clearly human-visible pedestrians. Together these quantify how often, and by how much, cooperation supplies information the following vehicle’s local perception misses.

The design choice to provide the LLM with pre-computed structured state fields rather than raw images is further validated by a vision model ablation. LLaVA-7B (via Ollama), the most widely adopted open-source multimodal model capable of local execution, was evaluated as an independent visual verification step. On the initial 30-pair subset on which it was run, LLaVA reported pedestrians = none in frames where YOLOv8 detected a pedestrian with high confidence, confirming that small open-source multimodal models are not yet reliable for fine-grained traffic detection in real dashcam imagery.

### 7.2. Limitations and Future Directions

The proposed architecture has several limitations that are identified for future work.

The BEBLID-based frame matching, inherited from prior work [19], produces match confidence values averaging 0.05–0.06 in urban scenes with dense vegetation, below the 0.13 quality threshold. As a result, the low-match-confidence safety rule is triggered on most pairs, correctly flagging the matching as uncertain; the pipeline still reaches a correct decision because the pedestrian-proximity rule dominates. Improving spatial matching robustness (through learned descriptors such as SuperPoint or GPS-anchored candidate filtering) is a priority for future iterations.

Model capacity affects reasoning quality. Early experiments with a small model (Qwen 2.5 1.5B) failed to identify pedestrian perception asymmetries in nearly all test cases, while larger production models performed reliably. Model availability also changed during development: the model used for the initial runs was deprecated by the provider and replaced, which also changed inference latency (Section 6.3). Characterizing the minimum model capability required for consistent cooperative reasoning, and doing so under a fixed model, remains an open question.

The current evaluation covers a single scenario type, the occluded pedestrian, extended to 60 frame pairs spanning the full occlusion-to-visibility transition and complemented by a negative normal-traffic set. The evaluation comprises manual human ground-truth annotation, ablation baselines (single-vehicle, rules-only, and LLM-only), a sensitivity analysis, and a negative test for false alarms. The ground-truth annotation was performed by one of the authors and was not blind to the system’s output; a larger study with multiple independent annotators and a formal agreement measure is future work. The main remaining limitation is scenario diversity: additional hazard types such as cyclists, sudden braking, and intersection encounters, and data from more vehicle pairs and conditions, are required before the results can be considered general. The modular architecture allows such extension without structural changes, and larger-scale validation across diverse scenarios is the primary direction for future work.

### 7.3. Deployment Considerations

The system is currently an offline cooperative-perception and decision-support framework, not a real-time in-vehicle system. Both dashcam streams are processed after recording: frames are matched, states are built, and the reasoning and safety layers are applied to the reconstructed joint state. This is appropriate for the present goal of demonstrating what cooperative reasoning can recover from real data, but a live deployment would face several additional constraints that are not addressed here.

First, the reasoning layer depends on a cloud-hosted language model. This introduces a dependency on network latency, connectivity, and provider availability. The latency measured in this work (from a few seconds to around twenty seconds per decision depending on the model) is too high for hard real-time control and would need to be reduced substantially, for example through a smaller on-device model. The pipeline already supports a local model backend, which removes the network dependency at the cost of reasoning quality; quantifying that trade-off is future work. Provider-side changes are a real risk: during this study one model was deprecated mid-development and had to be replaced. Data privacy is a further consideration, since dashcam footage may contain identifiable people and vehicles; a local-model configuration keeps raw imagery on the vehicle and mitigates this.

Second, the cooperation itself is reconstructed offline rather than exchanged over a live V2V link. A real deployment would add communication latency, packet loss, and bandwidth limits on top of the sensor-level time offset already handled here. In particular, network delay would add a variable lag between when the leading vehicle observes a hazard and when the following vehicle receives it, reducing the effective advance-warning margin reported in Section 6.6. Because the present work sends compact structured state rather than raw images, the bandwidth required is modest, but a full treatment of communication effects, including loss and out-of-order delivery, is left to future work.

Third, the safety layer provides a deterministic backstop but has been exercised only on the scenarios evaluated here. A systematic robustness study under corrupted GPS, missing or spurious detections, and contradictory vehicle states would be needed before any safety claim could be made for deployment. The current design already flags low-confidence matches and falls back to a cautious default on malformed reasoning output, which are steps toward fail-safe behavior but not a substitute for such a study.

## 8. Conclusions

This paper presented CoSafe, a five-layer cooperative perception and reasoning pipeline for V2V hazard detection operating on real-world dashcam data. The architecture integrates spatial–temporal vehicle matching, YOLOv8 object detection, structured state abstraction with explicit perception asymmetry encoding, LLM-based cooperative reasoning, and a deterministic safety validation layer.

The framework was evaluated on a real occluded-pedestrian scenario spanning 60 temporally aligned frame pairs, complemented by a 21-pair negative set with no hazard present and by manual human ground-truth annotation independent of the system’s detector. Without cooperation, the following vehicle detects the hazard in only 3 of 60 pairs, whereas the full pipeline reaches a detection rate of 1.00 with zero false alarms on the negative set. Cooperation provides an advance warning of at least 1.73 s (approximately 12 m of travel at the recorded speed) before the following vehicle’s own detector first registers the pedestrian. Measured against human annotation rather than the system’s own output, the Cooperation Gain is 0.533: in roughly half of the evaluated pairs the pedestrian was below the following vehicle’s reliable perception and the hazard information was available only through cooperation. The following vehicle’s local detector reaches a recall of only 0.107 against clearly human-visible pedestrians, quantifying the detector–human gap that cooperation compensates. The deterministic safety layer escalated the cases the LLM under-rated, confirming its role as a fail-safe backstop on top of the reasoning layer.

The key contributions cover system design and evaluation methodology: the modular pipeline design, the compact state abstraction schema, the perception asymmetry signal, and the Cooperation Gain metric, here reported against an independent human reference. The LLM serves as an interchangeable reasoning component: the framework is designed so that the underlying model can be substituted as newer or more capable models become available.

To the best of the authors’ knowledge, this is the first cooperative V2V perception framework operating on real monocular dashcam data without requiring LiDAR or dedicated V2V transceivers, complementing simulation-based work such as V2V-LLM and V2V-GoT.

Future work will focus on validation across diverse hazard types (cyclists, sudden braking, intersection encounters) and additional vehicle pairs and conditions, on characterizing the minimum LLM capability required for reliable cooperative reasoning under a fixed model, on improving the robustness of both spatial matching and on-board detection, and on modelling the effects of a live V2V communication channel (latency, packet loss, and bandwidth) on the advance-warning margin reported here.

## Figures and Tables

**Table 1:** Comparison of cooperative and LLM-based driving systems. Bold indicates the proposed CoSafe framework.

Criterion	DriveMLM	V2V-LLM	Alpamayo	Where2comm	CoSafe
Data	CARLA	OPV2V	nuScenes	OPV2V	**Real-world**
Sensors	4c+LiDAR	cam+LiDAR	4 cam	LiDAR	**2 dashcams**
Coop. V2V	No	Yes	No	Yes	**Yes**
Reasoning	MLLM	MLLM	VLA	–	**LLM+rules**
Explainable	Yes	Yes	Yes	No	**Yes**
Safety layer	No	No	No	No	**Yes**

**Table 2:** Pipeline configuration parameters.

Parameter	Value	Layer
ORB keypoints per frame	10,000	Layer 1
BEBLID descriptor size	64 values, scale 0.75	Layer 1
Lowe’s ratio test threshold	0.8	Layer 1
Match quality threshold	0.13	Layer 1
Trend window size *W*	5 frames	Layer 1
YOLO confidence threshold	0.35	Layer 2
Perception asymmetry conf.	0.70	Layer 3
Temporal context window	5 pairs	Layer 3
LLM temperature	0.1	Layer 4
Critical distance	5 m	Layer 5
High closing speed	5.5 m/s	Layer 5
Critical TTC	3 s	Layer 5
Warning TTC	6 s	Layer 5
Min match confidence	0.13	Layer 5
Pedestrian proximity	15 m	Layer 5
Critical $r_{\mathrm{kin}}$	0.9	Layer 5
Warning $r_{\mathrm{kin}}$	0.7	Layer 5

**Table 3:** Pedestrian visibility in the following vehicle’s view (author annotation over 60 pairs).

Label	Pairs	Description
no	6	Nothing separable from background
unclear	26	Progressively visible, below reliable-detection level
clear	28	Full silhouette, distinct contour

**Table 4:** Pipeline results for the occluded pedestrian scenario (60 frame pairs).

Metric	Value
Total frame pairs evaluated	60
YOLO pedestrian detection (Vehicle A)	60/60 (conf. 0.46–0.86)
YOLO pedestrian detection (Vehicle B)	3/60 (conf. 0.36–0.53)
Perception asymmetry (ϕ=1)	57/60
LLM risk assessment: HIGH	44/60
LLM risk assessment: MEDIUM	16/60
Safety layer overrides	76
low match confidence	60
pedestrian proximity	16
Final decision: HIGH/prepare_stop	60/60
Cooperation Gain (vs. human ground truth)	0.533

**Table 5:** Ablation results on the extended set (60 frame pairs).

Configuration	Detection Rate	LOW/MED/HIGH	Overrides
b_only	0.05	57/0/3	—
a_only	1.00	0/0/60	—
rules_only	1.00	0/0/60	—
llm_only	0.73	0/16/44	—
full	1.00	0/0/60	76

**Table 6:** Negative-set results (21 frame pairs, no pedestrian present).

Configuration	LOW/MEDIUM	False-Alarm Rate
b_only	21/0	0.00
a_only	21/0	0.00
rules_only	21/0	0.00
llm_only	19/2	0.00
full	19/2	0.00

**Table 7:** Sensitivity to the pedestrian-proximity threshold (combined positive and negative sets).

Proximity Threshold (m)	Detection Rate	False-Alarm Rate
8	0.83	0.00
12	1.00	0.00
15 (calibrated)	1.00	0.00
20	1.00	0.00

**Table 8:** Sensitivity to the match-confidence threshold (combined positive and negative sets).

Match-Confidence Threshold	Detection Rate	False-Alarm Rate
0.01	1.00	0.00
0.03	1.00	0.00
0.13 (calibrated)	1.00	0.00
0.25	1.00	0.00

**Table 9:** Sensitivity to the YOLO detection confidence threshold.

YOLO Threshold	B Detections	Advance Warning	Detection Rate
0.25	4/60	1.73 s	1.00
0.35 (calibrated)	3/60	1.73 s	1.00
0.50	2/60	1.73 s	0.983

## Data Availability

The source code and sample output files are available from the corresponding author upon reasonable request. All data were collected on public roads; identifiable information such as faces and license plates is not discernible at the resolution used, and no personal data were processed.

## Abbreviations

The following abbreviations are used in this manuscript:

ADAS	Advanced Driver Assistance Systems
BEBLID	Boosted Efficient Binary Local Image Descriptor
CG	Cooperation Gain
GPS	Global Positioning System
LLM	Large Language Model
MLLM	Multimodal Large Language Model
MPC	Model Predictive Control
NMEA	National Marine Electronics Association
ORB	Oriented FAST and Rotated BRIEF
TTC	Time-to-Collision
V2V	Vehicle-to-Vehicle
VLA	Vision–Language–Action
YOLO	You Only Look Once

## Appendix A. Chain-of-Thought Prompt

The complete prompt provided to the reasoning layer (Layer 4) is reproduced below. It enforces the three-phase PERCEIVE–FUSE–DECIDE structure, constrains the output to a fixed JSON schema, and includes explicit grounding rules that prevent the model from inventing hazards or overriding the structured state with unsupported visual claims. The placeholders {STATE_A}, {STATE_B}, and {TEMPORAL_CONTEXT} are filled at inference time with the structured state of each vehicle and the temporal context window. The prompt is designed to handle a broad range of cooperative driving scenarios (occlusion, braking, constrained corridors, traffic-light sharing, pedestrian and cyclist hazards, and others). The evaluation in this paper focuses on the occluded-pedestrian scenario; the remaining scenario types are supported by the same prompt structure but are not evaluated here, and are part of the broader validation planned as future work.

You are a cooperative driving safety reasoning module for an advanced driver assistance system (ADAS). You receive structured state information from two vehicles (A and B) that are on the same road and communicate via V2V. Your task is to analyze the situation cooperatively and generate a safety decision.

You MUST follow a strict 3-step Chain-of-Thought reasoning process:

STEP 1 - PERCEIVE:
Analyze what each vehicle perceives independently.

- What does Vehicle A see? What can it NOT see (occlusions)?
- What does Vehicle B see? What can it NOT see?
- Are there any pedestrians, traffic signs, or hazards detected?
- Use the temporal context to distinguish one-frame noise from persistent behavior.
- Use vehicle_speed_kmh, other_vehicle_speed_kmh, relative_speed_mps, closing_speed_camera_mps, distance_trend_slope, ttc_seconds, kinematic_risk_score, match_quality, both_sides_occupied, left_side_vehicle_count, and right_side_vehicle_count when they are present.
- If visual_description is present, use it only as supporting evidence, not as a replacement for the structured state.

STEP 2 - FUSE:
Combine the two perspectives through V2V cooperation.

- What new information emerges from cooperation that neither vehicle had alone?
- Is there any critical information that one vehicle has but the other lacks?
- Identify the cooperation value - what does V2V add here?
- Use dominant_trend, persistent_closing, min_ttc_window_s, distance_delta_window_m, avg_distance_trend_slope_window, and latest_match_quality from the temporal context when they are present.

STEP 3 - DECIDE:
Based on the fused understanding, generate a structured decision.

Scenarios you should handle include:

- Occlusion resolution (one vehicle sees a hazard hidden from the other)
- Emergency braking warnings (rapid distance closing)
- Constrained road corridor (vehicles present on both left and right sides)
- Traffic light state sharing (one vehicle can see the light, the other cannot)
- Pedestrian/cyclist hazards
- Emergency vehicle approach from behind
- Unexpected stopped vehicles - Normal traffic (no action needed - return LOW risk)

OUTPUT FORMAT:
Respond STRICTLY in valid JSON format with the following structure:

{

 "step1_perceive": {

  "vehicle_A_sees": "brief description",

  "vehicle_A_cannot_see": "brief description",

  "vehicle_B_sees": "brief description",

  "vehicle_B_cannot_see": "brief description"

 },

 "step2_fuse": {

  "new_information_from_cooperation": "brief description",

  "cooperation_value": "what V2V added - or ’none’ if no cooperation benefit"

 },

 "step3_decide": {

  "risk_level": "LOW|MEDIUM|HIGH|CRITICAL",

  "scenario_type": "occlusion|braking|constrained_road|traffic_light|pedestrian| emergency_vehicle|stopped_vehicle|normal|other",

  "action": "maintain|slow_down|prepare_stop|emergency_brake|change_lane|yield",

  "warning_to_A": "message for vehicle A driver (concise, imperative)",

  "warning_to_B": "message for vehicle B driver (concise, imperative, or ’none’)",

  "explanation": "brief technical explanation grounded in the input data",

  "confidence": 0.0

 }

}

IMPORTANT RULES:

- Your reasoning must be grounded strictly in the provided state data. Do NOT hallucinate facts.
- If a field is ’unknown’ or ’not_visible’, treat it as uncertain - do not assume.
- If occlusion is null, do NOT claim an occlusion or blocked view.
- If both_sides_occupied is true, you may describe a constrained corridor, but do NOT call vehicles parked unless the input states that explicitly.
- Prefer camera/GPS speeds and ttc_seconds for motion risk. Use distance_trend_slope as a trend-stability signal, not as the only evidence.
- Use scenario_type="constrained_road" when both sides are occupied and nearby vehicles meaningfully restrict maneuvering space.
- Use scenario_type="normal" only when there is no meaningful occlusion, no pedestrian hazard, no critical motion cue, and no corridor restriction.
- CRITICAL: If pedestrian_visible=true for ANY vehicle, risk_level CANNOT be "LOW" and scenario_type CANNOT be "normal". Use scenario_type="pedestrian" and at least risk_level="MEDIUM".
- CRITICAL: If Vehicle A detects a hazard that Vehicle B does NOT detect, this IS a perception asymmetry - the core value of V2V cooperation. In step2_fuse, cooperation_value MUST describe which vehicle sees the hazard and which does not.
- If one vehicle has pedestrian_visible=true at center_near position and distance_to_other_m < 15, use at least risk_level="HIGH" and action="prepare_stop".
- If frame_match_confidence is low or match_quality is low, lower confidence and mention uncertainty in the explanation, but do not invent hazards.
- If visual_description conflicts with structured fields, trust the structured fields.
- Do not infer a stopped vehicle, traffic-light state, parking lot, or intersection control unless directly supported by the inputs.
- For ’normal’ scenarios, return risk_level="LOW" and action="maintain".
- Warnings must be concise (under 20 words) and in imperative form.
- Explanation must reference at least two specific fields from the input.

NOW ANALYZE THE FOLLOWING STATES:
Vehicle A state: {STATE_A}
Vehicle B state: {STATE_B}
Temporal context: {TEMPORAL_CONTEXT}

Respond with the JSON only, no additional text before or after.

## References

[1] World Health Organization (2023). Global Status Report on Road Safety 2023.

[2] Jia D., Lu K., Wang J., Zhang X., Shen X. (2016). A Survey on Platoon-Based Vehicular Cyber-Physical Systems. IEEE Commun. Surv. Tutor..

[3] Cui E., Wang W., Li Z., Xie J., Zou H., Deng H., Luo G., Lu L., Zhu X., Dai J. (2025). DriveMLM: Aligning Multi-Modal Large Language Models with Behavioral Planning States for Autonomous Driving. Vis. Intell..

[4] Mao J., Ye J., Qian Y., Pavone M., Wang Y. A Language Agent for Autonomous Driving. Proceedings of the First Conference on Language Modeling.

[5] Sima C., Renz K., Chitta K., Chen L., Zhang H., Xie C., Beißwenger J., Luo P., Geiger A., Li H. DriveLM: Driving with Graph Visual Question Answering. Proceedings of the European Conference on Computer Vision.

[6] Wen L., Fu D., Li X., Cai X., Ma T., Cai P., Dou M., Shi B., He L., Qiao Y. DiLu: A Knowledge-Driven Approach to Autonomous Driving with Large Language Models. Proceedings of the International Conference on Learning Representations.

[7] Nie M., Peng R., Wang C., Cai X., Han J., Xu H., Zhang L. Reason2Drive: Towards Interpretable and Chain-Based Reasoning for Autonomous Driving. Proceedings of the European Conference on Computer Vision.

[8] Zhang Y., Liu J., Xu C., Hang P., Sun J. (2025). LeAD: The LLM Enhanced Planning System Converged with End-to-End Autonomous Driving. arXiv.

[9] Nazar A.M., Selim M.Y., Gaffar A., Qiao D. (2025). Situational Perception in Distracted Driving: An Agentic Multi-Modal LLM Framework. Front. Artif. Intell..

[10] Wang Y., Luo W., Bai J., Cao Y., Che T., Chen K., Chen Y., Diamond J., Ding Y., Ding W. (2025). Alpamayo-R1: Bridging Reasoning and Action Prediction for Generalizable Autonomous Driving in the Long Tail. arXiv.

[11] Wang Y., Jiao R., Zhan S.S., Lang C., Huang C., Wang Z., Yang Z., Zhu Q. Empowering Autonomous Driving with Large Language Models: A Safety Perspective. Proceedings of the LLM Agent Workshop at the International Conference on Learning Representations (ICLR).

[12] Hu Y., Fang S., Lei Z., Zhong Y., Chen S. (2022). Where2comm: Communication-Efficient Collaborative Perception via Spatial Confidence Maps. Proceedings of the Advances in Neural Information Processing Systems (NeurIPS).

[13] Xu R., Xiang H., Tu Z., Xia X., Yang M.H., Ma J. V2X-ViT: Vehicle-to-Everything Cooperative Perception with Vision Transformer. Proceedings of the European Conference on Computer Vision.

[14] Chiu H.K., Hachiuma R., Wang C.Y., Smith S.F., Wang Y.C.F., Chen M.H. (2026). V2V-LLM: Vehicle-to-Vehicle Cooperative Autonomous Driving with Multimodal Large Language Models. Proceedings of the IEEE International Conference on Robotics and Automation (ICRA).

[15] Xu R., Xia X., Li J., Li H., Zhang S., Tu Z., Meng Z., Xiang H., Dong X., Song R. V2V4Real: A Real-World Large-Scale Dataset for Vehicle-to-Vehicle Cooperative Perception. Proceedings of the IEEE/CVF Conference on Computer Vision and Pattern Recognition.

[16] Chiu H.K., Hachiuma R., Wang C.Y., Wang Y.C.F., Chen M.H., Smith S.F. V2V-GoT: Vehicle-to-Vehicle Cooperative Autonomous Driving with Multimodal Large Language Models and Graph-of-Thoughts. Proceedings of the IEEE International Conference on Robotics and Automation.

[17] Cui J., Tang C., Holtz J., Nguyen J., Allievi A.G., Qiu H., Stone P. (2025). Towards Natural Language Communication for Cooperative Autonomous Driving via Self-Play. arXiv.

[18] Hu S., Fang Z., Fang Z., Deng Y., Chen X., Fang Y. (2024). AgentsCoDriver: Large Language Model Empowered Collaborative Driving with Lifelong Learning. arXiv.

[19] Beti I.A., Herghelegiu P.C., Caruntu C.F. (2024). Architectural Framework to Enhance Image-Based Vehicle Positioning for Advanced Functionalities. Information.

[20] Rublee E., Rabaud V., Konolige K., Bradski G. ORB: An Efficient Alternative to SIFT or SURF. Proceedings of the International Conference on Computer Vision (ICCV).

[21] Suárez I., Sfeir G., Buenaposada J.M., Baumela L. (2020). BEBLID: Boosted Efficient Binary Local Image Descriptor. Pattern Recognit. Lett..

[22] Lowe D.G. Object Recognition from Local Scale-Invariant Features. Proceedings of the 7th IEEE International Conference on Computer Vision.

[23] Beti I.A., Herghelegiu P.C., Amarandei C.M., Caruntu C.F. Cloud Architectural Design for Image-Based Vehicle Positioning in Traffic Management. Proceedings of the 28th International Conference on System Theory, Control and Computing (ICSTCC).

[24] Beti I.A., Herghelegiu P.C., Caruntu C.F. Enhanced Frame-Matching Algorithm for Image-Based Vehicle Positioning Using Segmented Regions of Interest. Proceedings of the 2025 International Conference on Control, Automation and Diagnosis.

[25] Xie E., Wang W., Yu Z., Anandkumar A., Alvarez J.M., Luo P., Ranzato M., Beygelzimer A., Dauphin Y., Liang P., Vaughan J.W. (2021). SegFormer: Simple and Efficient Design for Semantic Segmentation with Transformers. Proceedings of the Advances in Neural Information Processing Systems (NeurIPS).

[26] Cordts M., Omran M., Ramos S., Rehfeld T., Enzweiler M., Benenson R., Franke U., Roth S., Schiele B. The Cityscapes Dataset for Semantic Urban Scene Understanding. Proceedings of the IEEE Conference on Computer Vision and Pattern Recognition (CVPR).

[27] Jocher G., Chaurasia A., Qiu J. (2023). Ultralytics YOLO.

[28] Popescu F., Stanescu D. (2026). AICEBERG: A Novel Agentic AI Framework for Autonomous Radio Monitoring, Compliance and Governance Based on LLM, MCP, and SCPI in Smart Cities. Smart Cities.

